# Digital Health in Parkinson’s Disease and Atypical Parkinsonism—New Frontiers in Motor Function and Physical Activity Assessment: Review

**DOI:** 10.3390/jcm14124140

**Published:** 2025-06-11

**Authors:** Manuela Violeta Bacanoiu, Ligia Rusu, Mihnea Ion Marin, Denisa Piele, Mihai Robert Rusu, Raluca Danoiu, Mircea Danoiu

**Affiliations:** 1 Sport Medicine and Physiotherapy Department, University of Craiova Romania, Craiova 200500, Romania; manuela.bacanoiu@edu.ucv.ro (M.V.B.); denisa.piele@edu.ucv.ro (D.P.); mihai.rusu@edu.ucv.ro (M.R.R.); mircea.danoiu@edu.ucv.ro (M.D.); 2 Faculty of Mechanic, University of Craiova Romania, Craiova 200500, Romania; mihnea.marin@edu.ucv.ro; 3 Sanocare Medical Center Craiova Romania, Craiova 200500, Romania; danoiuraluca@yahoo.ro

**Keywords:** digital health technologies, Parkinson’s disease, neurodegenerative disorders, physical activity

## Abstract

In addition to axial motor complications such as abnormal posture, instability, falls, and gait variability, neurodegenerative diseases like Parkinsonian syndromes include executive dysfunction, Parkinson’s disease dementia, and neuropsychiatric symptoms. These motor disorders significantly affect mobility, quality of life, and well-being. Recently, physical activity of various intensities monitored both remotely and face-to-face via digital health technologies, mobile platforms, or sensory cues has gained relevance in managing idiopathic and atypical Parkinson’s disease (PD and APD). Remote monitoring solutions, including home-based digital health assessments using semi-structured activities, offer unique advantages. Real-world gait parameters like walking speed can now be continuously assessed with body-worn sensors. Developing effective strategies to slow pathological aging and mitigate neurodegenerative progression is essential. This study presents outcomes of using digital health technologies (DHTs) for remote assessment of motor function, physical activity, and daily living tasks, aiming to reduce disease progression in PD and APD. In addition to wearable inertial sensors, clinical rating scales and digital biomarkers enhance the ability to characterize and monitor motor symptoms. By reviewing recent literature, we identified emerging trends in quantifying and intervening in neurodegeneration using tools that evaluate both remote and face-to-face physical activity. Our findings confirm that DHTs offer accurate detection of motor fluctuations and support clinical evaluations. In conclusion, DHTs represent a scalable, effective strategy for improving the clinical management of PD and APD. Their integration into healthcare systems may enhance patient outcomes, support early intervention, and help delay the progression of both motor and cognitive symptoms in aging individuals.

## 1. Introduction

Parkinsonism represents a group of neurodegenerative disorders characterized by tremor, muscle rigidity, bradykinesia, and postural instability. It is estimated that in the coming decades, approximately over 10 million individuals could be affected by PD or atypical Parkinsonism, making it the fastest-growing neurological disorder [1]. Motor dysfunction, or Parkinsonism, is a hallmark of PD, manifesting in both its early and advanced stages [2].

The four cardinal motor symptoms of PD—tremor, bradykinesia, rigidity, and postural instability—can be managed through pharmacological treatments [3]. Tremors in PD are categorized into two main types: rest tremors, which occur when muscles are relaxed, and action tremors, which appear during voluntary movements. Action tremors are further classified into postural impairment, occurring when a person holds a limb against gravity, and kinetic tremors, emerging during voluntary activities such as writing or eating. Idiopathic Parkinson’s disease (IPD) is the most common cause of rest tremor, though it can also contribute to action tremors [4]. Along with postural instability and gait difficulties, this neurodegenerative disorder also leads to an increased risk of falls as the disease progresses, further compromising mobility and independence.

Currently, the clinical diagnosis of Parkinson’s disease is often seen as inadequate, as it primarily relies on clinical criteria. This makes it difficult to achieve an early diagnosis of Parkinson’s disease and accurately and promptly differentiate it from atypical Parkinsonism. Additionally, other commonly acknowledged limitations include the short duration of clinical evaluations, which may not fully capture daily fluctuations in motor and cognitive dysfunctions, the subjectivity in clinical assessments, and the unreliability of patient self-reporting [1]. Recent advancements in digital health technologies have led to the emergence of user-friendly wearable devices capable of continuously monitoring motor fluctuations. Assessing and tracking the impact of motor severity on mobility and executive function of Parkinson’s disease is challenging due to its heterogeneous nature. Remote monitoring solutions can be implemented through home-based assessments using smartphones, tablets, wearable accelerometers, or even machine learning, as well as through specialist visits. These assessments rely on semi-structured activities, each offering specific advantages. In contrast, digital health technologies, such as body-worn sensors, enable the continuous and quantitative measurement of walking speed and clinically relevant gait characteristics (digital mobility outcomes) in real-world settings [4]. Due to the progressive nature of the disease, individuals with Parkinson’s disease (PD) gradually experience increasing difficulty in performing daily activities and a corresponding loss of autonomy. Over time, this leads to greater dependence on others for support with daily living, placing significant demands on caregivers [5]. Our study aims to evaluate the motor aspects of PD but also the impact of physical activities quantified using technological devices capable of recording and analyzing objective data on the motor abilities of individuals with Parkinson’s disease (PD) and their progression, as well as to highlight the need for using digital health systems correlated with clinically relevant evolving characteristics collected digitally and remotely. On the other hand, our study recommends motor telerehabilitation through using wearable sensors and other digital technology devices, which can enhance effectiveness, accessibility, and long-term outcomes.

## 2. Methods of the Literature Review

To recognize relevant research, we browsed the PubMed electronic database for all studies using the following keywords in different combinations: “digital health technologies”, “Parkinson’s disease”, “neurodegenerative disorders”, and “physical activity”. The original articles, clinical trials, pilot studies, or report cases in full text across a 5 year period were considered in our study. We considered 260 full papers, and after applying inclusion criteria, we analyzed 36. Non-original research formats such as literature reviews, systematic reviews, meta-analyses, editorials, commentaries, and letters to the editor were excluded. Additional articles were found in the reference lists of relevant publications. Only papers in English were taken from reference lists. We only considered papers that focused on physical activity and employed wearable sensors placed in different regions of the body, online platforms, motor clinical tools, and digital biomarkers to demonstrate delaying worsening functional autonomy in mild or moderate PD and other neurodegenerative disorders. Our review focused exclusively on studies targeting elderly individuals (typically aged ≥60 years) diagnosed with idiopathic Parkinson’s disease or atypical Parkinsonian syndromes (e.g., multiple system atrophy, progressive supranuclear palsy, or fronto-temporal dementia). Articles involving participants with major comorbidities (e.g., cardiovascular disease, advanced diabetes, stroke, or severe psychiatric conditions) were excluded, as were studies centered on younger populations or unrelated neurodegenerative conditions. Studies not directly related to digital health applications for Parkinson’s disease or Parkinsonian disorders (e.g., those focused solely on pharmacological interventions without digital components) were excluded. The selection process was conducted in two stages. First, titles and abstracts were screened to determine relevance. Second, full-text articles were assessed for eligibility based on predefined inclusion and exclusion criteria. Data extraction included the study design, sample size, participant characteristics, type of digital intervention, targeted clinical outcomes (motor, cognitive, and quality of life), and main findings. The search strategy included Boolean operators (AND, OR) where applicable. Eligible sources included original research articles, randomized controlled trials, feasibility and pilot studies, case reports, cohort studies, and clinical trials evaluating remote or face-to-face interventions using digital tools. Relevant references from selected articles were manually screened for additional eligible studies. Eligible studies used technologies such as wearable sensors, smartphone applications, tele-rehabilitation platforms, virtual reality systems, or digital biomarkers. To ensure clinical relevance, only studies that assessed functional outcomes (e.g., gait, balance, motor symptoms, executive function, and autonomy) were retained. For each included study, we extracted key methodological and clinical data, including study design, sample size, participant demographics, type of digital intervention, duration of the intervention, outcome measures, and main results. However, the variation in study design and outcome measures highlights the need for standardized methodologies and larger-scale trials to confirm long-term benefits.

## 3. Results

### 3.1. Determinants and Tools for Motor Assessment, Health Technology Devices, and Digital Biomarkers on Typical and Atypical Parkinson’s Disease

The dysfunctions in PD patients vary, ranging from motor impairments to cognitive issues, presenting in diverse forms. In the early stages, the condition manifests as tremors, but as the disease progresses, bradykinesia, postural disorders, and gait, balance, and coordination problems begin to emerge. For a complete assessment, the PD patient should follow precise kinematic tasks, all driven synergically, as in daily life they do not operate in isolation. The motor tasks can be generically grouped as comprehensive motor assessment scales, mobility and gait assessment tests, physical activity and autonomic function tests, and balance and confidence scales. All these indicators are used for PD and APD and are presented below in Table 1.

#### 3.1.1. Digital Health Technologies Role in PD and Atypical PD

Data collection involved specific stages and adhered to clearly defined time periods requiring a combination of wearable sensors, mobile applications, and motion-capture technology tools to evaluate motor symptoms for PD patients. Based on the predefined objectives of the studies, the devices used for the symptomatic assessment of patients with PD can be grouped into five distinct subcategories as follows: Wearable Sensors for Motion and Gait Analysis, Digital and Tablet-Based Motor Assessments, Advanced Motion Capture and AI-Enhanced Analysis, Neuroimaging and Clinical Evaluation, and Data Collection and Analysis.

##### Wearable Sensors for Motion and Gait Analysis

Motor symptoms were continuously monitored using wrist-worn tri-axial accelerometers (e.g., GENEActive, Fitbit Sense, Empatica E4, and the PKG™ system) to assess tremor, bradykinesia, and dyskinesia [5,6,7]. Gait analysis was performed using in-sole wearable sensors, the Quantitative Timed Up and Go (QTUG) system, and the IMU-based foot sensors, which provided objective measurements of mobility and fall risk [8,9,10]. Additional motion-tracking was conducted with the Opal-APDM system and Garmin Vivosmart 4, both of which measured stride length, step variability, and postural stability [11,12,13].

##### Digital and Tablet-Based Motor Assessments

Participants completed motor function tests using a smart tablet, which included assessments of tablet-based neurocognitive features [14]. Fine motor control and handwriting analysis were evaluated using a capacitive pen, while the Roche PD mobile application v2 and ALLFTD-mApp were employed for real-world symptom tracking [15,16,17]. Additionally, the mKinetikos system and TelePark tablet app provided remote monitoring capabilities [18,19].

##### Advanced Motion Capture and AI-Enhanced Analysis

Clinical, imaging, genetic, and transcriptomic data from patients with recently diagnosed Parkinson’s disease were analyzed using machine learning and deep learning algorithms. The findings revealed that the cerebrospinal fluid P-tau/α-synuclein ratio, along with atrophy in specific brain regions, may serve as relevant biomarkers for identifying distinct disease progression subtypes. Molecular analysis further uncovered genetic modules associated with each subtype, offering new insights into the development of personalized therapeutic strategies for Parkinson’s disease [20].

A motion-capture application was utilized to assess movement patterns, supplemented by AI-driven analysis through the Generalized linear mixed-effects model (GLMN) [21]. The Advanced Parkinson’s Disease Monitoring Device (adPMD) and Three-Dimensional Pose Tracker for Gait Test (TDPT-GT) system further contributed to data acquisition, integrating machine learning models for symptom progression analysis [3,22].

##### Neuroimaging and Clinical Evaluation

Brain imaging was conducted using SPECT tomography to assess dopamine transporter function [23]. Standardized clinical evaluations, including timed motor assessments, were performed using a stopwatch to measure reaction times and mobility performance [24]. The MR-005 system was employed to capture detailed motion characteristics [13].

##### Data Collection and Analysis

Raw sensor data were processed and analyzed using MaxQDA software for qualitative assessments, while quantitative data were extracted using the eHealth platform [7,19,25]. Additional wearable-based monitoring was conducted via the Ipsilon platform, the Oura Ring, and FlexiForce A401 for grip strength measurements [7,26]. The Adafruit Mini Motor Disc 1201 was integrated into the study to assess sensorimotor response in selected tasks [26]. A synthesis of these studies is presented in Table 2.

##### 3.1.2. Digital Biomarkers—Impact on PD and Atypical PD

A comprehensive evaluation of Parkinson’s disease symptoms requires the integration of multiple biomarkers that capture motor impairments and movement variability decline. Motor function was assessed using several frequency- and variability-based measures. The average value of the root mean square acceleration (aRMS) was employed to quantify movement irregularities and tremor intensity, providing an essential indicator of motor dysfunction [1]. To ensure the reliability of assessments, statistical and predictive metrics were incorporated. The Intraclass Correlation Coefficient (ICC) determined the consistency of repeated motor and cognitive measurements, reinforcing data reliability. Lastly, percentage daily times in motor states (PDTs) were used to evaluate dyskinetic state periods in order to forecast disease progression, supporting a more personalized and data-driven approach to symptom monitoring [3]. The Gini Index provided an additional statistical measure of movement irregularity, offering insights into symptom severity [26]. The Composite Physical Function Index (CPF) and Fall Risk Estimate (FRE) were utilized to analyze movement frequency patterns, particularly in relation to tremors and gait disturbances [9,24]. The Frailty Estimate (FE) was used to evaluate the complexity of movement, helping to predict the potential of fall occurrences [9].

The Baseline Tremor Index (BL) served as a reference point for motor function, enabling longitudinal comparisons while the Fluctuation Index (FI) was applied to measure the progression of each body position and its impact on motor behavior [1]. By integrating these diverse biomarkers, the study established a multidimensional framework for PD assessment, ensuring a comprehensive understanding of disease progression while facilitating individualized patient monitoring.

### 3.2. The Impact of Physical Activity Monitored Through Digital Health Tools on Only People PD

Additionally, physical rehabilitation programs utilizing digital health technologies can help monitor and improve postural stability and gait difficulties. For this reason, it is important to evaluate the role of physical activity and how to quantify this role.

#### 3.2.1. Activity Daily Living (ADL) Quantified by Digital Health Tools

Digital health tools can quantify daily activities through various methods, using devices and applications that monitor essential health and well-being parameters. There are some examples of daily activities that can be measured and analyzed. For instance, one study evaluates the validity of a wearable accelerometer-based digital Parkinson’s Motor Diary (adPMD) for tracking motor fluctuations in patients with advanced Parkinson’s disease (PD). By repurposing the Parkinson’s Kinetigraph (PKG^®^) to classify motor states (Off, On, and Dyskinetic), the study compares adPMD data to clinical diary assessments. The findings indicate moderate validity in detecting Off and Dyskinetic states, though temporal agreement between adPMD and clinical ratings is limited. Adjusting individual thresholds improves dyskinetic state detection. So, these results highlight the potential of adPMD for real-world monitoring but also underscore the need for refinement to enhance clinical reliability. The obtained results demonstrated moderate validation for estimating On, Off, and Dyskinetic states, with values of the intraclass correlation coefficient (ICC).

Agreements improved after individualizing adPMD for dyskinesia detection, with a median Cohen’s k range between 0.25 and 0.41 [3].

The feasibility and acceptability evaluation of a personalized digital program called KEEP (Knowledge, Exercise Efficacy, and Participation), designed to promote physical exercise among individuals recently diagnosed with Parkinson’s disease (PD), highlights an effective early-stage, non-pharmacological intervention that is easy to implement on a large scale. The intervention group, composed of individuals diagnosed with PD for less than one year, followed the KEEP program for eight weeks. The control group received standard care. The intervention was perceived as useful, accessible, and relevant. Participants reported a better understanding of the importance of exercise in managing PD. The feasibility was assessed by analyzing recruitment and follow-up rates, dropout rates, and the consistency of data collection regarding physical activity and inactivity. To avoid potential recording bias (e.g., not wearing the accelerometer for an entire day), consistent monitoring was emphasized. The effectiveness of the KEEP program was evaluated using both motor and scales. KEEP stands out by objectively measuring physical activity levels using wrist-worn accelerometers, reducing the self-reporting bias often seen in subjective activity assessments. Furthermore, the program supports the development of personalized behavioral habits through the acquisition of evidence-based knowledge delivered by healthcare professionals [6]. Another article, using the same equipment, such as wrist-worn devices via Parkinson’s KinetiGraph, which provides a continuous measure of movement parameters, was demonstrated to mitigate bradykinesia or dyskinesia through the scores of scales such as BKS, DKS, PSS2, MNSQ, or PDQ-8 [19] (Table 2).

#### 3.2.2. Physical Activity Type—Movement of Hands Related to Digital Technologies Tools

Hand movements associated with the use of digital technologies—such as smartphones, tablets, keyboards, and wearable devices—represent a distinct category of physical activity that can provide valuable insights into motor function, particularly in populations with neurodegenerative disorders like PD (Table 2).

These interactions often involve fine motor control, rhythmicity, and coordination, which can be monitored through sensors embedded in the devices or through external wearables (e.g., wrist accelerometers). Analyzing hand-based digital interactions enables passive, real-time assessment of motor performance in natural settings. Such data can be leveraged to detect subtle changes in dexterity, tremor, or bradykinesia, contributing to the development of digital biomarkers for early diagnosis and disease progression monitoring.

In accordance with motor scales MDS-UPDRS, H&Y, and Parkinsonian motor syndrome, researchers have discussed digital biomarkers such as motor activity (aRMS) and the tremor index (BL) based on using wrist-worn assessment systems through continuous recording as the first step or active tests as the second step. The passive continuous recording phase complements active UPDRS tests by addressing time limitations and enabling continuous monitoring of motor features. The active phase maintains a connection to current gold standards, focusing on bradykinesia and rest tremors through MDS-UPDRS motor tests using a wrist-worn accelerometer, which records pronation and supination movements. The novel aspect of this method is the combination of multiple parameters during passive continuous recording, which helps distinguish healthy subjects from those with Parkinsonian motor syndrome. In this context, smartphones or tablets that deliver Roche PD Mobile v2 emerge as a promising digital solution for the remote assessment of motor function in individuals with PD. Applications utilize built-in smartphone sensors to capture and analyze motor and vocal performance through a series of structured active tests. These tests are conducted on both sides of the body and involve drawing shapes on the touchscreen, alternating taps on virtual buttons to assess finger dexterity, and rotating the hand holding the phone to evaluate hand-turning speed and range. The strongest numerical associations were observed for sensor-derived features related to bradykinesia, particularly those obtained from the hand-turning and finger-tapping tests (the patient must keep tapping an index finger on a table until the examiner instructs the patient to stop). These measures showed higher concordance with clinical evaluations, especially on the more affected side of the body. These findings highlight the variable sensitivity of digital assessments depending on the specific motor domain being evaluated. It represents a valuable tool in the context of telemedicine and digital health, supporting personalized and continuous care for patients with PD [16].

#### 3.2.3. Physical Activity and Wearable Devices for the Assessment of Gait, Postural Control, and Functional Mobility in Parkinson’s Disease Subtypes

Regular physical activity, exercise, and adequate nutritional status are essential for slowing the progression of symptoms and maintaining physical function in Parkinson’s Disease. A six-month study evaluated a mobile health technology (mHealth)-based follow-up program focusing on the self-management of exercise and nutrition after interdisciplinary rehabilitation. The primary outcome showed improvement in physical capacity (6MWT), while secondary outcomes included improvements in nutritional status, health-related quality of life (HRQOL), physical function, and exercise adherence. Physical activity such as natural walking, lower- or moderate-intensity walking, short walking bouts, “walking for exercise”, or real-world walking speed (RWS) in people with PD and motor fluctuations was evaluated using accelerometer wearable devices based on a digital Parkinson’s Motor Diary (adPMD) using Kinetigraph or ankle devices—SAM. The motor functions of patients with Parkinson’s disease (PD) were evaluated during different motor fluctuation states: “Off” state, “On” (activated) state, and the Dyskinetic period.

The study aims to validate a new digital marker, namely fall count, using a previously reported fall risk assessment algorithm that incorporates PIGD clinical scores, gait and balance assessments, and a novel mathematical approach based on elastic net and ensemble regression analyses, which combine multiple machine learning models [9]. To assess the feasibility, proof of concept, and preliminary clinical outcomes associated with delivering a self-guided digital walking intervention based on music and the principles of Rhythmic Auditory Stimulation (RAS) for individuals with Parkinson’s disease in a naturalistic setting, twenty-three individuals with PD used the digital intervention independently for four weeks. They completed five weekly 30 min unsupervised walking sessions outdoors, with music-based cues. The intervention progressed autonomously based on real-time gait detection. The feasibility of independent use was assessed by examining adherence, safety, and participants’ experience. The proof of concept for the intervention was evaluated by examining spatiotemporal gait quality values, daily moderate-intensity walking minutes, and daily steps [13]. Improvements in gait parameters and motor functions were monitored using digital biomarkers, which were collected from assessments conducted on devices such as smartphones, neurocognitive tablets, smartwatches, or stopwatches. Parkinson’s patients need personalized medicine, which is essential for optimizing treatments. To implement personalized medicine using machine learning, relevant features must be identified, as the performance of algorithms largely depends on the quantity and quality of extracted data. In this context, another study collected nearly 275 features from various assessments: PRO (Patient-reported outcomes) questionnaires (e.g., PDQ-39), functional movement evaluations (e.g., TUG, STS, and 6MWT), and digital biomarkers (e.g., tablet-based assessments). The goal was to identify significant features for classifying PD patients and enhance the ability to analyze these objective data for a better understanding of patients’ true capabilities. Decision tree classification revealed new important values for digital functional assessments, emphasizing the need to expand methods of measuring movement abnormalities (such as akinesia, rigidity, and tremor) that cannot be effectively evaluated through traditional paper-based tests [27]. The features that captured postural tremors and rest tremors showed strong correlations, highlighting their sensitivity in detecting motor impairments. In another study, the feasibility, patient satisfaction, adherence, and usability of a mHealth system utilizing Android smartphones were assessed. The main goal of the system was to provide continuous and objective monitoring of patients’ health and functional mobility [16,18]. In another study, 56 patients with PD were monitored over five consecutive days using a commercial smartwatch, with daily step counts recorded and averaged across all days, weekdays, and weekends. Reliability was assessed using the intraclass correlation coefficient (ICC), standard error of measurement (SEM), Bland–Altman analysis, and minimal detectable change (MDC). The findings indicated that a minimum of four days of monitoring is necessary to achieve acceptable reliability (ICC ≥ 0.84; SEM < 10%). Additionally, no significant differences were found in step counts across different days or between weekdays and weekends. These results support the use of daily step count as a robust measure of ambulatory activity in PD, with implications for real-world monitoring, prevention strategies, and rehabilitation planning [12]. Another study aimed to identify the temporospatial gait abnormalities in patients with idiopathic normal pressure hydrocephalus (iNPH) and Parkinson’s disease, as well as to distinguish between the two conditions by analyzing fluctuations in body positioning dynamics and identifying their specific characteristics. The research utilized a non-invasive motion-capturing application (TDPT-GT) on an iPhone, which generates 30 Hz coordinates from 27 points on the body. Gait was recorded in a 1 m diameter circle for three groups: iNPH patients, PD patients, and a healthy control group. Significant differences in mean slopes were tested using one-way ANOVA, and multiple comparisons were performed between each pair of groups. The results revealed significant differences between the patient groups and controls across all body positions, with patients consistently showing lower absolute values. The system demonstrated the ability to measure full-body movement and temporal variations during gait. Abnormal fluctuations in the movement of both the upper and lower body may significantly contribute to gait and balance disturbances in patients, thereby highlighting the impact of these abnormalities on functional mobility in both conditions [22]. Immersive Virtual Reality (IVR) has shown promise as a tool for assessing and predicting fall risk in individuals with Parkinson’s Disease (PD). Given the high fall risk and the association of reaction times with postural instability and disease progression, IVR-based reaction time tests were explored as potential predictors of falls. Another study demonstrated the feasibility of IVR interventions without adverse effects. Reaction times measured through IVR were significantly correlated with functional tests, as well as cognitive performance and patient age, but not with the initial PD symptoms or disease stage. These findings suggest that IVR could serve as a complementary tool for fall prevention, offering a simple and rapid method to predict fall risk in PD patients [28].

At the same time, we found that Integrated Gait Analysis (IGA) combined multiple gait parameters to assess balance, stride variability, and the risk of falls, while the Mobility Risk Score offered a predictive measure of mobility limitations, helping to estimate fall risk based on gait and balance metrics [10].

## 4. Discussion

Traditional clinical diagnosis, primarily based on subjective motor assessments, has variable accuracy, particularly in early-stage PD, leaving a substantial proportion of cases undiagnosed. Emerging biomarker-based approaches show promise for early detection, but their reliance on invasive procedures limits widespread adoption. In this context, advanced digital technologies, such as wearable devices and sensors, offer a promising alternative by generating continuous data streams relevant to PD monitoring. Consumer-grade wearable devices and sensors have the potential to improve estimates of Parkinson’s disease prevalence and surveillance indicators by providing broader access to screening tools [34]. Our study focused on participants diagnosed with PD and APD at various clinically confirmed stages, collecting high-dimensional data from multiple sensors during a multidomain assessment battery. Assessments were conducted using consumer-grade smartwatches, wearable inertial sensors, smartphones, tablet-based neurocognitive features, or trunk-mounted devices with timing functionality. Remote-monitored wearable devices and sensors provide an accurate and reliable method for evaluating PD at a population level in real-world environmental conditions. Digital biomarkers, wearable sensors, and smart devices could provide objective and sensitive measures for monitoring Parkinson’s disease, with continuous monitoring being a promising marker for detecting prodromal PD. Evaluating motor signs and extracting key features to differentiate early PD from healthy controls can be effectively achieved using these technologies. As such, there are major challenges in the precise and early identification of the disease, highlighting the need for more effective and objective diagnostic methods. Currently, the clinical diagnosis makes it challenging to establish an early diagnosis and perform an accurate and timely differential diagnosis between Parkinson’s disease and other forms of Parkinsonism [1]. Therefore, the data collected using wearable technologies monitored all cardinal manifestations of Parkinson’s disease (PD) and Atypical Parkinsonism (APD), including bradykinesia combined with rigidity, rest tremor, and other motor fluctuations. Continuously captured data related to gait, balance, fine motor skills, and other mobility patterns are crucial for detecting subtle changes in motor performance that might not be evident during a clinical visit, enabling early intervention and more personalized treatment protocols [3,13,21,33]. Several studies have compared clinical observations recorded in patient home diaries with data obtained from wearable accelerometer-based systems, such as the Digital Parkinson’s Motor Diary (adPMD), to monitor motor fluctuations in individuals with advanced Parkinson’s disease. The findings indicate moderate validity in detecting Off and Dyskinetic states, although the temporal agreement between adPMD outputs and clinical assessments remains limited. Notably, adjusting individual thresholds improves the accuracy of dyskinesia detection. These results underscore the potential of Parkinson’s Kinetigraph for real-world motor symptom monitoring, while also highlighting the need for further refinement to enhance its clinical reliability [3,4]. Gait variability and turning times, as well as balance and mobility measurements that reflect fall risk, have been assessed using inertial sensors placed on the wrists, the lumbar region, both feet, and vibrating socks (FlexiForce A401) on PD and APD patients. Receiver Operating Characteristic (ROC) analysis can help determine an optimal test threshold that accurately identifies individuals at high risk of falling (high sensitivity) while minimizing false positives among those without risk (high specificity). Additionally, the correlation of risk factors such as the PIGD score, SMDT performance, muscle fatigue, and cerebrospinal fluid biomarkers has been used to estimate the likelihood of fall onset [11,13,23,26,34]. Other studies, through the analysis of spatio-temporal parameters and macro- or micro-gait characteristics using inertial measurement units (IMUs) or generalized linear mixed models (GLMMs), have demonstrated that gait-focused physiotherapy (GPT) leads to greater improvements in gait performance compared to standard physiotherapy. These findings, obtained via wearable sensors, were particularly relevant in patients with multiple system atrophy (MSA) or progressive supranuclear palsy (PSP) and highlighted associations between real-world digital gait measurements and abnormal fatigue progression [10,21]. Various types of digital health technologies—such as smartphones [16,18,35], smartwatches, and wearable devices [12,18,24,36]—have been investigated for their ability to assess motor and symptoms of Parkinson’s disease (PD). The reliability and validity of these mobile applications lie in their capacity to capture multiple active and passive measures related to dyskinetic state functioning. Tasks such as the electronic Symbol Digit Modalities Test (eSDMT), the Spiral Writing and Clock Test (SWCT), and unidimensional and extended multifunctional tasks have been optimized to improve the detection and remote monitoring of both motor and symptoms.

Other researchers have employed digital questionnaires, semi-structured topic-guided interviews, and digital survey tools in combination with home-based digital dance programs to monitor activities of daily living (ADL), walking, turning, balance, finger and hand movements, and coordinated dance-related motor activities [5,17,19,25,30,37]. Immersive virtual reality (IVR) has shown promise as a tool for assessing and predicting fall risk in individuals with Parkinson’s disease (PD), given the high risk of falls and the association between reaction time and postural instability.

Our study demonstrated the potential of a digital technology-based approach—utilizing various inertial sensors, smartphones, smartwatches, online platforms, neurocognitive tablets, immersive virtual imaging, and structured interviews—to objectively monitor physical activities of varying intensities, both in-person and remotely, in individuals with Parkinson’s disease (PD) and atypical Parkinsonian disorders (APD).

Our study demonstrated that digital health technologies (DHT) have the potential to objectify assessments traditionally conducted using gold-standard motor and clinical tools and can contribute to the clinical staging of Parkinson’s disease (PD) subtypes. For individuals with Parkinson’s disease, digital phenotyping represents a valuable tool for continuous motor and symptom monitoring, the improvement of gait parameters and walking bouts, personalized treatment adjustments, and alleviating caregiver burden. Conversely, for older adults without PD, smart and engaging environments can facilitate the maintenance of an active lifestyle and contribute to the prevention of age-related functional decline [7,38]. Daily step count can serve as a valuable indicator of real-world ambulation in individuals with Parkinson’s disease, yet the minimum number of monitoring days required for reliable estimation using commercial smartwatches had not been previously established. Future research should emphasize the integration of digital technologies to enhance early diagnosis, refine clinical staging, and improve the monitoring of motor and cognitive fluctuations in neurodegenerative conditions such as PD and APD and disease progression. IVR-based reaction time tests have also been explored as potential predictors of falls. The integration of digital technologies into the monitoring and management of Parkinson’s disease presents promising opportunities for personalized care, enhanced adherence to physical activity, and effective remote intervention. Wearable devices, mobile applications, and digital biomarkers enable objective, continuous, and ecologically valid assessment of motor functions, supporting the early detection of functional decline and real-time treatment adjustments. In this context, digital interventions may become essential components of a comprehensive, individualized care strategy for people living with Parkinson’s disease [39,40].

Furthermore, in accordance with other studies, we find that features derived from the devices, particularly arm swing, the proportion of time with tremor, and finger tapping, differed significantly between individuals with early PD and age-matched controls and had variable correlation with traditional assessments [41]. Hill et al. confirmed in 2022 that sensor data from digital health technologies (DHTs) used in clinical trials provide a valuable source of information because of the possibility to combine datasets from different studies with other data types and reuse them multiple times for various purposes [5].

## 5. Conclusions

This study emphasizes the potential of digital health technologies to enhance the monitoring and clinical evaluation of Parkinson’s disease (PD) and atypical Parkinsonian disorders (APD). The integration of wearable sensors, smartphone applications, virtual reality environments, and tablet-based cognitive tools has shown promise in detecting motor features with greater objectivity, flexibility, and remote accessibility. The strengths of the study include the multimodal approach, the correlation between digital biomarkers and established clinical tools, and the feasibility of remote, continuous monitoring.

However, several limitations persist, including moderate temporal agreement between digital and clinical assessments, limited improvements in mood-related outcomes, and challenges related to device heterogeneity and user accessibility. Despite these constraints, the findings support the role of digital phenotyping in early diagnosis, clinical staging, and personalized management.

Future work should focus on refining algorithmic thresholds, increasing user-friendliness, and validating digital tools in larger longitudinal cohorts to better inform disease progression, treatment response, and caregiver support strategies. However, the variation in study design and outcome measures highlights the need for standardized methodologies and larger-scale trials to confirm long-term benefits.

## Figures and Tables

**Table 1:** Health digital technologies related to PD and APD.

Motor Tools	Instruments	Digital Biomarkers
MDS-UPDRS Motor Examination section (part 3) of the Unified Parkinson’s Disease Rating Scale	Wrist-worn tri-axial accelerometer	aRMS
H&Y Hoehn & Yahr	Smart tablet	BL
TUG Time-Up-and Go	Capacitive pen	ICC
9 NHTP Nine-Hole Peg Test	adPMD	CPF
SPDDS Self-assessment Parkinson’s Disease disability score	PKG^TM^	FE
PIGD Postural instability/gait difficulty	In-sole wearable sensors-based gait analysis	FI
UMSARS Unified Multiple System Atrophy rating scale	Tablet-based neurocognitive features	FRE
BBS Berg balance scale	MaxQda software	Gini index
STS/5TSTS Sit to stand;	Stop-watch	PDTs
6MKT six-minute walk test	OPAL-APDM	Mobility risk score
SEE Self-efficacy for exercise	Gene active	Wisconsin Card
MOEES Multifactorial outcome expectation for exercise scale	Fibit sense	
KEPA-PD Knowledge on exercise and physical activity in PD questionnaire	Empatica E4	
RPAQ Recent physical activity questionnaire	Oura ring	
G-SAP Gait-specific attentional profile	Ipsilon	
CRS Clinical rating scales;	eHealth platform	
PAM Physical activity monitoring	Wearable sensors on the feet-IMU	
IPAQ International physical activity questionnaire	Accelerometer spectra	
FOG-Q Freezing of gait questionnaire	GLMN	
SCOPA-AUT Scales for outcomes in Parkinson’s disease autonomic	AI	
10-MWT 10-m Walk Test	SPECT tomograph	
TDPT-GT Three-Dimensional Pose Tracker for Gait Test	QTUG	
SWST Stand-Walk-Sit-Test	Roche PD mobile application v.2	
TUG-C-IVR Cognitive IVR	Android smartphone	
GABS-B Gait and Balance Scale Part B	ALLFTD-mApp	
ABC Activity-specific Balance Scale	MR-005 system	
	mKinetikos system	
	TGPT-GT motion- capture application	
	Garmin Vivismart 4	
	FlexiForce A401	
	Adafruit mini motor disc 1202	
	Exergaming	
	IVR	
	TelePark tablet app.	
	REMAP-Real-word	

Instruments/tools: adPMD: Wearable accelerometer-based digital PD Motor diary, Off, On, dyskinetic state; PKG: Parkinson’s, Kinetigraph; OPAL-APDM: three inertial sensors—both feet, lumbar region, wrists; Fitbit sense, Empatica E4: smart watch; Ipsilon: interactive task-based digital cognitive assessment tool; Oura Ring: smart ring of wrist skin, IMU: inertial measurement unit; GLMN: Generalised linear mixed-effects model; AI: Binary classification-machine learning; QTUG: Kinesis Health technologies-wearable inertial sensors; MaxQda software: semi-structured topic-guide interviews digitally recorded and transcribed verbatim, ALLFTD-mApp: ARTFL-LEFFTDS Longitudinal Frontotemporal Lobar Degeneration mobile app; IVR: Immersive virtual reality.

**Table 2:** Summary of studies’ findings.

Authors	Participants	Characteristics	Age/Gender	Tools	Conclusions
Löhle M., et al. (2023) [3] Waking, ADL	63 participants PD with motor fluctuations 46% men	VALIDATE-PD study synopsis (disease and symptom duration, duration of fluctuation, clinical phenotype, reported motor complications, PD medications), n = 40-German subcohort, n = 23-Swedish subcohort	About 66 years	Wearable accelerometer-based digital PD Motor diary Kinetograph	- Monitoring of motor fluctuations in PD patients, but their validity compared to clinical standards is unclear - Moderate validity for estimating daily time in Off and Dyskinetic states - Improved agreements observed after individualizing adPMD thresholds for dyskinesia detection
Kirk C., et al. (2023) [4] Walking	88-PD, 111-healthy control group	ICICLE Gait study (monitoring gait, cognitive decline and falls in early PD-36 months) Real-world gait assessment protocol	58–81 years	Wearable devices, Accelerometer	- Monitoring through RWS of the fluctuating impairments of PD - Complement monitoring of mobility in PD, using real-world walking and walking bout duration.
Templeton J. M., et al. (2022) [27] Walking, Neurocognitive speaking	50-PD 50-control group (CP)	Functional test: e.g., motor, speech, memory, executive function), Multifunctional test	50–85 years	Tablet-based neurocognitive features	- In early-stage PD, perceived deficits in memory and executive function were 21.6% lower than sensor-based scores, meaning disparity between subjective perception and objective measurements - Enhancing significantly elevated perceptions of deficits in executive and behavioral functions in advanced stages of PD compared to early-stage patients.
Prime M., et al. (2020) [24] Walking	104 PD, 43-female, 61-male	PIGD phenotype-67 participants, TG phenotype-37, 30 participants with assistive devices	68 ± 9 years	Stop-watch	- Enhancing the efficiency of the 360° turn test in relation to the PIGD phenotype, with high sensitivity regarding the number of steps and time - Regarding reactive postural control and tandem walking, no differences were observed between the two subtypes - Reduced performance observed in the number of strides and time to turn during the 360° Turn Test, as well as the duration of One-leg stance.
Shah V.V., et al. (2020) [11] Walking	29-PD 27-HC	43 digital biomarkers of mobility (e.g., pitch at initial contact, at toe off, gait speed, stride, turn duration length, swing, cadence, double support, sagittal range of motion, step in turn).	67.66 ± 5.27 years	Opal-APDM (three inertial sensors): Both feet, Lumbar region, Wrists	- Turning and gait variability are best predictors for monitoring PD - Turning emerged as the most important mobility domain - Increases in turning duration in individuals with PD and fall risk are a form of fall protection and facilitation of balance control.
Torrado J.C., et al. (2022) [7] Walking, ADL	First study: 15-PD/15 Helgetun residents-CG Second study: 90 dyads from Digi Park, 90 participants from Helgetun branch (residents and people on the waiting list)	Active Aging framework cyclic study for four years, Digi Park Branch, Helgetun branch, Device monitoring and human data collection: “device level” and “human level”	Older adults	Smartwatch-Fitbit sense, Smartwatch-Empatica E4, Smart ring-Oura Ring-wrist skin, Ipsilon-interactive task-based digital cognitive assessment tool, eHealth platform	Digital phenotyping can investigate clinical diagnosis, symptom tracking, and treatment response in PD patients Through explicable Artificial Intelligence (XAI), it is possible to evaluate decisions, modify parameters, and verify biases.
Greene B.R., et al. (2021) [9] Walking	1057 participants, three cohorts: 1. healthy community-dwelling older adults (1015) 2. PD1-longitudinal study-Order of Saint Francis (15), 3. PD2-cross sectional study, less impaired than PD1(27).	Control group-first cohort, PD1-12.1 falls/participant for 24 weeks, through daily falls diaries PD2-0.37 falls/participant measured retrospectively, self-reported	61.3–74.4 years	Wearable inertial sensors (QTUG-Kinesis Health technologies)	Through a detailed kinematic assessment, it is possible to predict fall occurrences using two pre-existing trained models (the FRE model and the Mobility model), which have previously been evaluated for fall risk assessment.
Lipsmeier, F., et al. (2022) [16] Walking, ADL, Movement of hands, Neurocognitive speaking	316 participants early-stage PD	PASADENA study22	-	Roche PD mobile application v2, Android smartphone, Smartwatch	- Quantifying reliability and validity of remote at-home application for the severity of motor issues in early PD
Zajac J.A., et al., 2023 [13] Walking	23-PD	Clinical Study (30-min sessions of unsupervised, overground walking with music-based cues)	About 66 years	MR-005 system	- Monitoring the walking capacity of PD patients through gait and functional parameters - Creating correlation analysis of gait parameters with PD clinical severity - Significantly enhancing life quality for PD patients via the use of online rehabilitation program.
Bouça-Machado R., et al., 2021 [18] Walking, Movement of hands	20-PD	Clinical Study (daily survey, three weekly active tests, performance of monthly in-person clinical assessment)	60.8 ± 11.2 years	mKinetikos system	- Monitoring and establishing the correlations of the clinical outcomes with the mKinetikos scores - Quantification of the mKinetikos scores evolution by daily, weekly, monthly, and base time monitoring - Integrating online interventions for gait, freezing, and postural instability with beneficial results.
Bianchini E et al., 2023 [12] Walking	56-PD patients	Clinical study investigating the minimum number of days required to reliably estimate the average daily steps in PD patients	69.5 ± 7.8 years	Garmin Vivosmart 4, smartwatch	- Accurately measuring the total daily steps with the aim of investigating the minimum number of days needed for a reliable estimation - Comparing the distribution of daily steps for both working and weekend days without demonstrating any significant differences.
Klaver E. C. et al., 2023 [26] Walking	40-PD	Clinicat study (Monitoring gait for recent history of disabling or regular FOG ON dopaminergic state OFF dopaminergic state)	66 (60–74) years	Vibrating socks motor control unit, pressure sensor (FlexiForce A401 pressure sensor) and vibration motor (Adafruit Mini Motor Disc 1201)	- Monitoring the average percent time frozen of uncued gait for both ON and OFF dopaminergic states - Monitoring the cueing strategies and establishing connections between them and the number of freezing episodes or the spatiotemporal gait parameters
Nuic D. et al., 2024 [28] Walking	50 patients 25-PD 25-Control Group	Clinical Study	About 69 years	Home-based, tailored, exergaming training system	- Monitoring the evolution of the functional mobility for PD patients through changes in the SWST parameter - Longitudinal assessment and comparison regarding between-group differences for the changes between baseline and after-training performance based on specific scales - Active exergaming training improved clinical gait and balance disorders scores as well as postural gait kinetics
Campo-Prieto P. et al., 2023 [29] Walking	26 PD 10-Fallers 16-Non-Fallers	Clinical Study (exploring the feasibility of reaction time tests performed in IVR as predictors of falls)	69.73 ± 6.32 years	IVR Immersive virtual reality	- Monitoring cut-off points reaction time, TUG test, and TUG-Cognitive test - Assessing the functionality of each group when using IVR - Monitoring and connection establishment between reaction time and cognitive impairment as well as functionality and disease progression
Mammen J.R. et al., 2023 [30] Walking, Movement of hands, Neurocognitive speaking	40-PD	Clinical Study 12-month multicenter monitoring PD symptoms and disease progression in people with early, untreated PD	-	WATCH-PD	- Monitoring the symptom mapping for each PD patient - Assessing the tasks in order to establish the most relevant symptoms for the majority of PD patients - Enhancing standardized assessment with the purpose of improving understanding of relevance for diverse contexts of use.
Bendig J. et al., 2022 [19] Waking, Movement of hands	18-PD	Observational Study	69 (37–86) years	eHealth solutions, including 3D-camera system, Wearable system (PDMonitor mobile app.), Tablet app. (TelePark tablet app)	- Initial assessment of motor and cognitive functioning based on specific scales - Monitoring the motor performance in accordance with predefined tasks - Monitoring and correlating the used system with age and motor scores or with cognition status - Enhancing the eHealth intervention in an adaptive manner for a specific segment of age and cognitive and motor functioning.
Alnes R.S. et al., 2023 [31] Walking	100-PD 50-Intervention Group 50-Control Group	Clinical Study-controlled trial protocol self-management exercise and nutritional protocol	≥40 years,	Garmin Vivosmart 4 (Mhealth system)	- Longitudinal digital follow-up based on physical capacity and nutritional status at baseline and three and six months - Monitoring the use of Mhealth system with the help of the activity tracker for comparing daily goals reached
Bek J. et al., 2022 [14] Walking	149-OA 178-PD	Clinical Study 12-month participation	50–89 years 47–88 years	Digital home dance program	- Monitoring and comparing the motor outcomes for both healthy and PD adults - Enhancing the use of digital home dance for its therapeutical and cost-effective provision.
Morgan C. et al., 2023 [32] Walking	24 participants, 12-PD 12-Control Group	Clinical Study (Sensor-based collected data for 5 days)	61.25 years 59.25 years	REMAP-REal-world Mobility Activities in Parkinson’s disease Wearable wrist-worn accelerometer, Skeleton	- Assessment of daily mobility - Monitoring the accelerometry readings for STS and non-STS activities - Comparing the collected results from healthy participants with those collected from PD patients.
Girnis J.L. et al., 2023 [33] Walking	82-PD	Clinical Study (cross-sectional analysis of low to moderate walking intensity)	67.5 ± 8.4 years	Accelerometer SAM on legs device (Step watch)	- Monitoring the number of steps and number of minutes per day of walking at various levels of walking intensity to categorize participants into 2 groups: more active or less active - Correlating the categories active/less active with the intensity and duration of the walking exercise
Geerlings A.D., et al. (2023) [5] ADL	504-PD 17-informal givers	Cross-sectional study with quantitative and qualitative (semi-structured interviews) approaches-PRIME-NL	50–70 years	Questionnaires	- Enhancing emotional stress of informal caregivers in trying to support the patient, who could suffer from diminished self-esteem or depression due to inactivity and the disease itself.
Santini S., et al. (2022) [25] ADL, Neurocognitive speaking	18 PD, 7-females 11-males	Digital cognitive rehabilitation (CTR) treatment, Face-to-face rehabilitation method, QUANT data, QUAL data	73.1 ± 4.8 years	Semi-structured topic-guide interviews digitally recorded and transcribed verbatim, MaxQda software	- Increasing efficacy and accessibility of online cognitive treatment adapted to PD for cognitive functions but not on their mood
Agley L., et al. (2024) [6] ADL	29-PD	8 weeks KEEP study: 6 interactive digital modules, 4 online live group discussions	67.3 ± 10.8 years	Wrist accelerometer (GENE active), Telephone	- Assessing the feasibility and acceptability of a co-designed digital intervention aimed at encouraging exercise and physical activity in individuals recently diagnosed with Parkinson’s disease - Using accelerometers to objectively monitor physical activity proved both feasible and effective, offering an alternative to self-reported activity measures, which can often be less reliable
Wang F., et al. (2022) [23] ADL	68-FoG group, 115-non-FoG group,	PPMI cohort, observational study, Five years	52.8–69.6 years	Siemens or General electric SPECT tomograph	- Correlating risk factors such as the PIGD score, muscle fatigue, or Abeta 42 levels in CSF with the onset of FoG.
Dominey T. et al., 2020 [8] ADL	166 PD patients FU = 71 NP = 78	Clinical Study (PKG™ monitoring for 3–6 months)	46–85 years 39–87 years	Parkinson’s KinetiGraph (PKG™), wrist-worn device that provides a continuous measure of movement	- Continuous monitoring of physiological PD patient status through Kinetograph recordings - Assessment of sleep quality (Sleep fragmentation, Daytime somnolence) through PDSS2 scale
Battista L., et al. (2024) [1] Movement of hands	59-PD 41-healthy controls	First step: Continuous recording, Second step: Active test	60–70 years	Wrist-worn tri-axial accelerometer	- Continuous monitoring and the integration of various characteristic motor features - Quantifying two core manifestations—bradykinesia and rest tremors—using specific motor tests from Part 3 of the MDS-UPDRS - Enhancing the ability to differentiate between healthy individuals and those with Parkinsonian motor syndrome

PD: Parkinson’s Disease; MDS-UPDRS: Motor Examination section (part 3) of the Unified Parkinson’s Disease Rating Scale; PIGD: Postural Instability and Gait Difficulties sub-score of MDS-UPDRS.

## Data Availability

No new data were created or analyzed in this study.
